# Evaluating Economic and Clinical Impacts of Anaemia Management Strategies: A Systematic Review of the Evidence From the UK Perspective

**DOI:** 10.1002/jha2.70124

**Published:** 2025-08-26

**Authors:** Hiro Farabi, Florian Tomini, Hayley Evans, Michael F. Murphy, Laura Green, Paula Dhiman, Gianluca Fabiano, Antony J. R. Palmer, Linda von Neree, Simon J. Stanworth

**Affiliations:** ^1^ Center for Evaluation and Methods, Wolfson Institute of Population Health Queen Mary University of London London UK; ^2^ NIHR Blood and Transplant Research Unit in Data‐Driven Transfusion Practice Nuffield Division of Clinical Laboratory Sciences Radcliffe Department of Medicine University of Oxford Oxford UK; ^3^ NHS Blood and Transplant Oxford University Hospitals NHS Foundation Trust and University of Oxford Oxford UK; ^4^ Blizard Institute Queen Mary University of London London UK; ^5^ NHS Blood and Transplant Barts Health NHS Trust London UK; ^6^ Nuffield Department of Orthopaedics Rheumatology and Musculoskeletal Sciences University of Oxford UK

**Keywords:** anaemia, blood transfusion, cost‐effectiveness, cost‐effectiveness, erythropoiesis‐stimulating agents, healthcare resource utilisation, patient blood management (PBM), restrictive transfusion strategy, UK healthcare system

## Abstract

**Background:**

Anaemia significantly affects health outcomes and quality of life. While blood transfusion remains a common intervention, alternative treatments, such as iron supplementation and erythropoiesis‐stimulating agents (ESAs), offer potential to mitigate transfusion‐associated costs. However, robust evidence on their cost‐effectiveness remains limited.

**Objective:**

This review assesses the cost‐effectiveness of anaemia treatments, aiming to inform UK healthcare policy and practice.

**Methods:**

A systematic review was conducted following PRISMA guidelines, identifying economic evaluations published between 2015 and 2025. Study quality was appraised using the Drummond checklist and NICE reference case criteria. Data were synthesised using the Hierarchical Decision Matrix framework.

**Results:**

Of 5496 records screened, 14 studies met inclusion criteria; 11 were included in the final synthesis, with three excluded due to low methodological quality. Restrictive transfusion strategies were cost‐saving (£35.50–£75 per patient), reduced red blood cell utilisation by ∼21%, shortened length of stay by 0.5 to 3 days, and yielded modest QALY gains (0.01 to 0.02). ESAs reduced transfusion risk (RR 0.61 to 0.87) but incurred substantial incremental costs (£1859–£3060) with limited evidence of QALY gains. Transfusion of fresher blood in ICU settings increased costs without a measurable clinical or economic advantage. Preoperative erythropoietin and ferric carboxymaltose reduced transfusion incidence but were high‐cost interventions with limited evidence on QALY gains. Patient Blood Management (PBM), particularly intravenous iron, was cost‐saving (£30.80–1166 saved per patient), reduced transfusion rates (RR 0.61), but with limited evidence on QALY gains.

**Conclusion:**

Restrictive transfusion thresholds and PBM interventions, especially intravenous iron, demonstrate favourable cost‐effectiveness and potential for NHS cost savings. In contrast, the cost‐effectiveness of ESAs remains uncertain due to high costs and limited utility evidence. Further research is needed to capture long‐term outcomes and generate UK‐specific economic data.

**Trial Registration:**

The authors have confirmed clinical trial registration is not needed for this submission.

## 1 Introduction

Anaemia remains a significant global health challenge, affecting an estimated 1.76 billion people—approximately 23.7% of the global population—with the highest burden observed among preschool children and women of reproductive age [1]. Iron deficiency is the leading cause, responsible for nearly half of all anaemia cases, though its contribution varies by region, population group, and the prevalence of infectious diseases [2]. Despite improvements in healthcare infrastructure and nutritional standards in many countries, the condition persists as a major cause of morbidity [1, 3].

Blood transfusion is a well‐established treatment for severe anaemia, offering rapid correction of haemoglobin levels and improved oxygen delivery to tissues [4, 5, 6]. However, transfusions are associated with non‐negligible risks and substantial healthcare costs. Reported complications include transfusion reactions (0.5%–3% incidence), alloimmunisation, iron overload and transmission of infectious agents such as hepatitis B, hepatitis C and HIV, although rare in high‐income settings [7, 8].

Transfusions are also limited by resource availability, and they also impose substantial costs on healthcare systems [9, 10]. In 2024, the average NHS cost of a red blood cell unit was £186.25, with administration adding £49 per unit, resulting in a total per‐unit cost exceeding £235 [11, 12]. As a result, there is a growing emphasis on cost‐effective and safer alternatives. These include iron supplementation, erythropoiesis‐stimulating agents (ESAs), nutritional strategies and other haemoglobin‐optimising interventions, particularly in settings such as perioperative care. When appropriately targeted, these alternatives may reduce reliance on transfusions, lower healthcare costs and minimise adverse outcomes [13]. However, these alternatives also pose important safety considerations. In particular, ESAs have been associated with increased thromboembolic risk and potential tumour progression in oncology populations, prompting regulatory warnings and necessitating careful patient selection [8, 14].

The full impact of anaemia treatments extends beyond clinical outcomes, encompassing both direct healthcare costs and indirect costs such as lost productivity and reduced quality of life. While the clinical efficacy of various anaemia interventions is well‐documented, their economic implications have received comparatively limited attention. The National Institute for Health and Care Excellence (NICE) addressed this gap in its 2015 guideline on blood transfusion (NG24), which included a review of economic evidence related to anaemia treatment [13]. The guideline stressed the importance of cost‐effectiveness, particularly in surgical settings, and encouraged the use of alternatives to transfusion when appropriate. However, it also acknowledged significant gaps in the economic evidence base, especially concerning the cost‐effectiveness of transfusion strategies themselves.

Despite the substantial resource implications of anaemia management, comprehensive economic evaluations comparing transfusions to alternative treatments remain scarce. Recent studies have underscored the need for more comprehensive cost‐effectiveness analyses to inform healthcare decision‐making, improve patient outcomes and facilitate efficient resource allocation. [15, 16] However, existing reviews have been largely narrative in nature, focusing primarily on methodological aspects of economic modelling rather than systematically evaluating outcomes across interventions [15]. This highlights the immediate need for a comprehensive review of recent economic evaluations to inform policy and guide clinical practice.

Our systematic review addresses the existing gaps in economic evaluations of anaemia treatments by systematically assessing both the economic and clinical impacts of various interventions, including blood transfusions. We incorporate the NICE economic evaluation checklist to ensure relevance to UK healthcare settings, focus on studies from Organisation for Economic Cooperation and Development (OECD) countries, including recent research, and adhere to NICE appraisal methods to identify pertinent literature. Building upon prior assessments, such as the NICE guideline on blood transfusion [13], this review aims to provide updated insights to inform clinical decision‐making and policy development.

## 2 Methods

### 2.1 Search Strategy and Inclusion/Exclusion Criteria

A comprehensive literature search was conducted in January 2023 and updated in February 2025. The search strategy was informed by a prior review undertaken by NICE [13] and adhered to the PRISMA (Preferred Reporting Items for Systematic Reviews and Meta‐Analyses) guidelines. The protocol was registered with PROSPERO (CRD42022346634) [17].

We included all published economic evaluations of interventions aimed at managing anaemia that involved or sought to avoid blood transfusion. Eligible study designs included randomised controlled trials (RCTs), cohort and observational studies, systematic reviews and meta‐analyses. Both full and partial economic evaluations were considered, including cost‐effectiveness analyses and budget impact analyses. Studies of all anaemia types and treatment modalities were eligible. Key health outcomes included quality‐adjusted life years (QALYs), mortality, number of blood transfusion units, transfusion relative risk and hospital length of stay.

Eligible studies were limited to those published in English from OECD countries between January 1, 2015, and January 31, 2025. Additional relevant studies identified from the NICE NG24 review were summarised separately. Databases searched included MEDLINE, Embase, CENTRAL, the Cochrane Library, EconLit, Web of Science, Scopus, the Transfusion Evidence Library, ClinicalTrials.gov and the WHO International Clinical Trials Registry Platform (ICTRP).

We excluded non‐comparative cost studies (e.g. cost‐of‐illness or cost descriptions without comparison), studies solely focused on non‐anaemia‐related transfusions, inherited anaemia or those lacking full‐text access. Letters, opinion pieces, conference abstracts and posters were also excluded.

### 2.2 Protocol Deviations

While the original protocol [17] for this review included a broader population of patients requiring transfusions, the selection and synthesis of studies focused on economic analyses involving patients with anaemia, consistent with the main aim of the review. Studies deemed to be of low methodological quality were retained in the review and documented in the quality assessment table. However, their findings were not included in the comparative synthesis if their design lacked sufficient comparative rigour (e.g. uncontrolled before–after studies or aggregate‐level outcomes), limiting their interpretability for policy‐relevant economic evaluation.

### 2.3 Study Selection

All identified references were imported into Covidence for systematic screening. Title and abstract screening was conducted independently by two reviewers (HF and SN), followed by full‐text review of potentially relevant articles. Studies were excluded at either stage based on predefined criteria, with justifications recorded. Discrepancies were resolved through discussion or adjudication by a third reviewer (FT).

### 2.4 Data Extraction

Data extraction was conducted independently by two reviewers (HF and FT) using a pre‐designed template within Covidence. Extracted information included study characteristics (e.g., year, country, authorship, clinical area and target population), as well as details of the economic evaluation (e.g., intervention, comparator, time horizon, discount rate, perspective and sensitivity analyses). Cost and outcome data included resource use (staff time, blood use, hospitalisation, medications), incremental cost‐effectiveness ratios (ICERs) and results from subgroup and sensitivity analyses. Given our focus on cost‐effectiveness and resource use outcomes (e.g. QALYs, transfusion rates, hospital stay), we prioritised outcomes that were consistently available and relevant to economic decision‐making, including incremental costs and QALYs, as well as changes in mortality, transfusion risk, units transfused and hospital days. Any disagreements were resolved by consensus.

### 2.5 Data Analysis

Extracted data were exported into Excel for analysis. All cost data were converted to 2023 GBP using OECD Purchasing Power Parity (PPP) rates [18]. If the study year was unspecified, the publication year was used for these adjustments.

Given the heterogeneity across anaemia types and intervention approaches, a direct comparison of cost‐effectiveness was not always feasible. To address this, we applied the Hierarchical Decision Matrix (HDM) [19], a decision‐support tool that enables transparent ranking of interventions based on cost and outcome combinations. Interventions were categorised into three groups—accept, review or reject, to assist in prioritising options based on economic value. Interventions were categorised into three groups—‘accept’, ‘review’ or ‘reject’, to assist in prioritising options based on economic value. For instance, an intervention was classified as ‘accept’ if it demonstrated both cost savings and improved outcomes (e.g., higher QALYs, reduced transfusion rates or shorter hospital stays), or as ‘review’ if it showed higher costs but improved outcomes, and as ‘reject’ if it involved both higher costs and similar or negative outcomes. The HDM offers a pragmatic and transparent way to compare interventions when formal economic synthesis is not feasible, and it may be particularly useful in the absence of consistent ICERs or QALY data. However, it simplifies complex analyses and does not incorporate uncertainty or quality of evidence, which should be considered when interpreting its classifications.

### 2.6 Quality Assessment

Quality assessment was conducted using Drummond's 10‐point checklist for economic evaluations—focusing on the rigour of design, methods and reporting (including uncertainty and sensitivity analysis) [20]—and the NICE Economic Evaluation checklist (focusing on the UK of policy relevance) [21] of each study. Two reviewers independently assessed each study, with discrepancies resolved through discussion. For the Drummond checklist, items were rated as ‘fully met’ (1 point), ‘unclear’ (0.5 points) or ‘not met/not applicable’ (0 points). Studies were categorised as high quality (scores of 8.0 or above), medium quality (scores of 6.0–8.0) or low quality (scores below 6.0). For the NICE checklist, each criterion was rated and averaged across relevant items to assess methodological limitations and UK applicability. Studies were classified as having low limitations (average scores of 8.0–10.0), medium limitations (scores of 6.0–8.0) or high limitations (scores below 6.0), and as having high, medium or low applicability based on the same thresholds. These assessments informed the inclusion of studies in the final synthesis and the interpretation of results.

## 3 Results

### 3.1 Study Selection

The initial search yielded 5515 records. After removing 19 duplicates, 5496 records were screened by title and abstract. Of these, 5195 were excluded. Full‐text retrieval was sought for 301 studies, with 243 assessed for eligibility.

**FIGURE 1:**
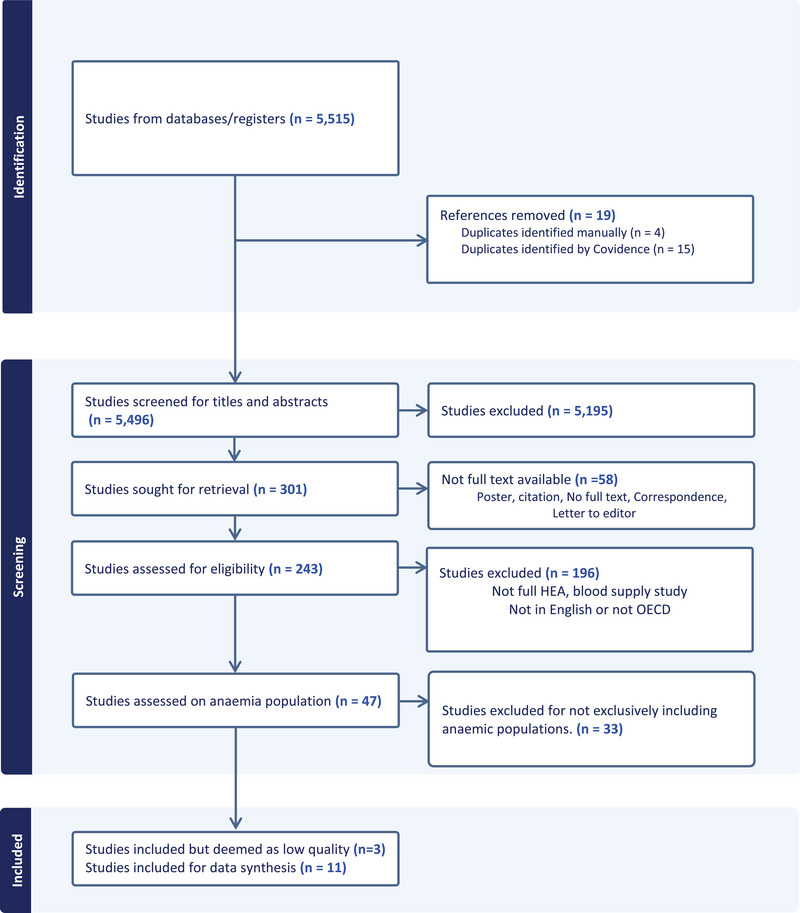
PRISMA diagram.

Overall, 232 studies were excluded because they did not meet the inclusion criteria. The main reasons for exclusion were lack of accessible full text or publication format (e.g. poster, citation, correspondence, letter to the editor) (*n* = 58), did not represent full health economic evaluations or focused on blood supply services (*n* = 196), or did not exclusively include anaemic populations (*n* = 33). In total, 14 studies were included in the review. Figure 1 outlines the full study selection process.

### 3.2 Quality Assessment

Fourteen studies were appraised using the Drummond checklist for economic evaluations [20]. Three studies [22, 23, 24] were excluded from further analysis due to lower methodological quality. Of the eleven remaining studies, one was rated as high quality (with a score greater than 8.0), demonstrating strong adherence to key evaluation criteria, while the others were medium quality, meeting most standards with some limitations. Full scoring details are in Tables 1, 2, 3.

**TABLE 1:** Drummond Checklist—scores per question for each of the initially selected studies

Study authors (Year)	1. Was a well‐defined question posed in an answerable form?	2. Was a comprehensive description of the competing alternatives given (i.e. who did what to whom, where, and how often)?	3. Was the effectiveness of the programme or services established?	4. Were all the important and relevant costs and consequences for each alternative identified?	5. Were costs and consequences measured accurately in appropriate physical units (e.g. hours of nursing time, etc)?	6. Were the cost and consequences valued credibly?	7. Were costs and consequences adjusted for differential timing?	8. Was an incremental analysis of costs and consequences of alternatives performed?	9. Was allowance made for uncertainty in the estimates of costs and consequences?	10. Did the presentation and discussion of study results include all issues of concern to users?	Score	Quality classification
Crathorne et al. (2016) (UK)	Yes	Yes	Yes	Can Not Tell	Can Not Tell	Can Not Tell	Yes	No	Yes	Yes	7.5	Medium Quality
Durand‐Zaleski (2022) (France & Spain)	Yes	Yes	Yes	Can Not Tell	Can Not Tell	Can Not Tell	Not Applicable	No	Yes	Yes	7.2	Medium Quality
Irving et al. (2019) (Australia)	Yes	Can Not Tell	Yes	Can Not Tell	Can Not Tell	Can Not Tell	Not Applicable	Yes	Yes	Yes	7.8	Medium Quality
Walsh et al. (2017) (UK)	Yes	Can Not Tell	Yes	No	Yes	Yes	Not Applicable	Yes	Yes	Yes	8.3	High Quality
Bedair et al. (2015) (USA)	Yes	Can Not Tell	Not Applicable	Can Not Tell	Yes	Yes	Not Applicable	No	Yes	Can Not Tell	6.1	Medium Quality
Sanal et al. (2023) (Turkey)	Yes	Yes	Yes	Can Not Tell	Can Not Tell	Can Not Tell	Yes	Not Applicable	Yes	Yes	6.0	Medium Quality
Basora et al. (2018) (Spain)	Yes	Yes	No	Can Not Tell	Can Not Tell	Can Not Tell	Not Applicable	Yes	Yes	Can Not Tell	6.7	Medium Quality
Trentino 2021 (Australia)	Yes	Yes	No	Can Not Tell	Yes	Can Not Tell	Not Applicable	Yes	Yes	Yes	7.8	Medium Quality
Meybohm et al. (2020) (Germany)	Yes	Yes	Not Applicable	No	Yes	No	Not Applicable	Yes	Yes	Yes	7.5	Medium Quality
Drabinski 2020 (Germany)	Yes	Can Not Tell	Not Applicable	No	Yes	Can Not Tell	Not Applicable	Yes	Yes	Yes	6.7	Medium Quality
Tatar et al. (2022) (Turkey)	Yes	Yes	Can Not Tell	Can Not Tell	No	Can Not Tell	No	Yes	No	Yes	4.5	Low Quality
Husk et al. (2024)	Yes	Can Not Tell	No	Can Not Tell	Can Not Tell	Can Not Tell	Not Applicable	Can Not Tell	Yes	Yes	6.1	Medium Quality
Linn et al. (2024) (USA)	Yes	No	No	Can Not Tell	Can Not Tell	Not Applicable	No	No	No	Can Not Tell	2.8	Low Quality
Czempik PF et al. (2024)	Yes	Can Not Tell	No	No	Can Not Tell	Can Not Tell	Not Applicable	Can Not Tell	Yes	Yes	5.6	Low Quality

*Note*: Criteria were rated as ‘Yes—Fully Met’ (1 point), ‘Can Not Tell’ (0.5 points), or ‘No—Not Met’ (0 points). Scores were averaged over the number of applicable criteria. Studies were categorised as ‘High Quality’ (average ≥8/10 points), ‘Medium Quality’ (average 6–7.5 points), or ‘Low Quality’ (average <6 points).

**TABLE 2:** Applicability (relevance to specific review questions and the NICE reference case)

Study authors (Year)	1.1 Is the study population appropriate for the review question?	1.2 Are the interventions appropriate for the review question?	1.3 Is the system in which the study was conducted sufficiently like the current UK context?	1.4 Is the perspective for costs appropriate for the review question?	1.5 Are non‐direct health effects on individuals excluded?	1.6 Are all future costs and outcomes discounted appropriately?	1.7 Are quality‐adjusted life years (QALYs), derived using NICE's preferred methods, or an appropriate social care‐related equivalent used as an outcome?	Score* (out of 1.0)	Applicability classification
Crathorne et al. (2016) (UK)	Yes	Yes	Yes	Yes	Yes	Yes	Yes	1.00	High applicability
Durand‐Zaleski (2022) (France & Spain)	Yes	Yes	Partly/Can Not Tell	No	Yes	NA	Yes	0.75	Medium applicability
Irving et al. (2019) (Australia)	Yes	Partly/Can Not Tell	Partly/Can Not Tell	No	Yes	NA	Yes	0.67	Medium applicability
Walsh et al. (2017) (UK)	Yes	Yes	Yes	Partly/Can Not Tell	Yes	NA	Yes	0.92	High applicability
Bedair et al. (2015) (USA)	Yes	Yes	Partly/Can Not Tell	No	NA	NA	No	0.50	Low applicability
Sanal et al. (2023) (Turkey)	Yes	Yes	No	No	Yes	NA	No	0.5	Low applicability
Basora et al. (2018) (Spain)	Yes	Yes	Partly/Can Not Tell	No	Yes	NA	No	0.58	Low applicability
Trentino et al. (2021) (Australia)	Yes	Yes	Partly/Can Not Tell	No	Yes	NA	No	0.58	Low applicability
Meybohm et al. (2020) (Germany)	Yes	Yes	Partly/Can Not Tell	Partly/Can Not Tell	No	NA	NA	0.60	Medium applicability
Drabinski et al. (2020) (Germany)	Yes	Yes	Partly/Can Not Tell	Partly/Can Not Tell	NA	NA	No	0.60	Medium applicability
Katherine (2024) (USA)	Yes	Yes	Partly/Can Not Tell	Partly/Can Not Tell	Yes	NA	Partly/Can Not Tell	0.75	Medium applicability

*Note*: Criteria were rated as ‘Yes—Fully Met’ (1 point), ‘Partly/Can Not Tell/Can Not Tell’ (0.5 points), or ‘No—Not Met’ (0 points). Scores were averaged over the number of applicable criteria. Studies were categorised as ‘High Applicability’ (average ≥0.8 points), ‘Medium Applicability’ (average 0.6–0.75 points), or ‘Low Applicability’ (average <0.6 points).

Abbreviation: NA, not applicable.

**TABLE 3:** Limitations (relevance to specific review questions and the NICE reference case)

Study authors (Year)	2.1 Does the model structure adequately reflect the nature of the topic under evaluation?	2.2 Is the time horizon sufficiently long to reflect all important differences in costs and outcomes?	2.3 Are all important and relevant outcomes included?	2.4 Are the estimates of baseline outcomes from the best available source?	2.5 Are the estimates of relative intervention effects from the best available source?	2.6 Are all important and relevant costs included?	2.7 Are the estimates of resource use from the best available source?	2.8 Are the unit costs of resources from the best available source?	2.9 Is an appropriate incremental analysis presented, or can it be calculated from the data?	2.10 Are all important parameters whose values are uncertain subjected to appropriate sensitivity analysis?	2.11 Has no potential financial conflict of interest been declared?	Score* (out of 1.00)	Limitations Classification
Crathorne et al. (2016) (UK)	Partly	Yes	Yes	Yes	Yes	Partly	Partly	Yes	NA	NA	No	0.78	Medium limitations
Durand‐Zaleski (2022) (France & Spain)	Yes	Partly	Yes	Partly	Partly	Partly	Partly	Partly	Yes	Partly	Yes	0.64	Medium limitations
Irving et al. (2019) (Australia)	Yes	Partly	Yes	Partly	Partly	Partly	Partly	Yes	Yes	Partly	Yes	0.73	Medium limitations
Walsh et al. (2017) (UK)	NA	Partly	Yes	Yes	Yes	Yes	Partly	Partly	Yes	unclear	No	0.70	Medium limitations
Bedair et al. (2015) (USA)	Yes	Partly	NA	NA	NA	Partly	Partly	Partly	Yes	Partly	No	0.56	Medium limitations
Sanal et al. (2023) (Turkey)	Yes	Partly	Partly	NA	Partly	Partly	No	Partly	Yes	Partly	No	0.45	High limitations
Basora et al. (2018) (Spain)	Partly	Partly	No	Partly	Partly	Partly	Partly	Partly	Yes	unclear	Yes	0.50	High limitations
Trentino et al. (2021) (Australia)	Unclear	Partly	No	Partly	Partly	Partly	Partly	Partly	Yes	Partly	Yes	0.50	High limitations
Meybohm et al. (2020) (Germany)	Yes	Partly	NA	NA	NA	Partly	Partly	Partly	No	Yes	No	0.50	High limitations
Drabinski et al. (2020) (Germany)	Yes	Partly	NA	NA	Partly	Partly	Partly	Partly	NA	Partly	No	0.50	High limitations
Katherine (2024) (USA)	Yes	Partly	No	No	No	Partly	Partly	Yes	Yes	Partly	Yes	0.50	High limitations

*Note*: *Criteria were rated as ‘Yes—Fully Met’ (1 point), ‘Partly’ (0.5 points), or ‘No—Not Met’ (0 points). Scores were averaged over the number of applicable criteria. Studies were categorised as ‘Low Limitations’ (average ≥0.8 points), ‘Medium Limitations’ (average 0.6–0.75 points), or ‘High Limitations’ (average <0.6 points).

Abbreviation: NA, not applicable.

### 3.3 Study Characteristics

Among the selected studies for further data analysis, four conducted a cost‐utility analysis (CUA) [25, 26, 27, 28], three conducted a cost‐effectiveness analysis (CEA) [29, 30, 31], one conducted both CUA and CEA [32], two used a cost‐minimisation analysis (CMA) [33, 34], and one performed a cost‐benefit analysis (CBA) [35] (see Table 4). These studies were conducted in several countries, including the UK [25, 28], USA [26, 33], France and Spain [32], Australia [27, 30], Turkey [31], Spain [29], and Germany [35, 39].

**TABLE 4:** Overview of Included Studies: Key Characteristics and Cost‐Effectiveness Results.

Study authors ID & study country	Blood management intervention / Speciality	Target population of the study	Type of full economic evaluation	Study design	Source/Data collection	Intervention	Perspective/ Time Horizon / Currency/ Discounting	Main results for Incremental Effectiveness Ratio (ICER)	Study's conclusions
Crathorne et al. (2016) (UK)	Erythropoietin / Anaemia (PBM)	Patient with cancer treatment‐induced anaemia (CIA)	Cost‐Utility Analysis	Model‐based economic evaluation (decision‐analytic model)	Literature review	**Intervention**: SAs (epoetin alfa, beta, theta and zeta and darbepoetin alfa) **Comparator**: Supportive care, defined as adjusting cancer treatment, RBCT and iron supplementation	**Perspective**: NHS and Personal Social Service **Time horizon**: Lifetime **Currency**: £ Pound /2014/15 **Discount rate**: 3.5% per annum	Incremental cost‐effectiveness ratios (ICERs) for ESA treatment compared with no ESA treatment from £19,429 to £35,018 per QALY gained.	EPO improves haematological response and reduces RBC transfusion needs, enhancing health‐related quality of life (HRQoL). However, its uncertain effects on side effects and survival raise doubts about the cost‐effectiveness of ESAs if survival remains unchanged.
Bedair et al. (2015) (USA)	Erythropoietin / Surgery (Hip/Lower limb arthroplasty)	Patients undergoing primary THA or TKA with preoperative haemoglobin < 13 g/dL	Cost‐Minimisation Analysis	Observational study with cost‐minimisation analysis	Own study data from Department of Orthopaedics, Massachusetts General Hospital, Boston, MA, USA	**Intervention**: At least one dose of EPO preoperatively (EPO group) for patients with a pre‐operative haemoglobin of<13 g/dL **Comparator**: No‐erythropoietin alpha (EPO)	**Perspective**: Not explicitly mentioned (HC provider assumed) **Time Horizon**: Not explicitly mentioned (short‐term) **Currency**: USD / 2012	NA	EPO reduced the need for postoperative transfusions in high‐risk patients undergoing THA and TKA; however, it was not found to be cost‐effective.
Trentino et al. (2021) (Australia)	Patient Blood Management/Surgery (Colorectal)	PBM Adults booked for elective colorectal surgery	Cost‐Effectiveness Analysis	Observational cohort with statistical modelling	Clinical data were sourced from the Western Australia Patient Blood Management Data System	**Intervention**: Preoperative screening **Comparator**: Not screened	**Perspective**: Health service perspective **Time Horizon**: Short‐term **Currency**: Australian dollars/ 2019 **Discount**: Not Applicable	Unadjusted results: AU$ −36,716 per RBC units avoided Multivariable regression model: AU$ −31,094 per RBC units avoided Propensity score weighting = AU$ −33,123 per RBC units avoided	Pre‐operative screening for anaemia and low iron stores in patients undergoing elective colorectal surgery led to a reduction in the number of red cell units transfused, while also being more cost‐effective than not screening.
Meybohm et al. (2020) (Germany)	Patient Blood Management / Surgery (General elective)	Patients with IDA (Iron‐deficiency anaemia) undergoing elective surgery	Cost‐Benefit Analysis	Modelled analysis using meta‐analysis and secondary data	Meta‐analysis prospective observational study	**Intervention**: implementation of a multimodal PBM programme **Comparator**: No multimodal PBM	**Perspective**: Healthcare system perspective **Time Horizon**: Hospital stay duration **Currency**: Euros **Discount rate**: Not Applicable (Costs and outcomes were time‐independent)	Hypothetical implementation of PAMs in Germany in 2015 would have resulted in substantial cost savings of approximately €1029 million.	The cost‐utility analysis from an NHS perspective showed no significant differences in costs or outcomes between fresh blood and standard‐aged blood. As a result, the findings suggest that there is no justification for favouring fresh blood over standard‐aged blood based on differences in quality of life, longevity, or cost considerations.
Walsh et al. (2017) (UK)	Red blood cells / Intensive care (mechanical ventilation)	Critically ill patients aged ≥ 18 years (≥ 16 years in Scotland) expected to require mechanical ventilation for ≥ 48 h and requiring a first RBC transfusion during the first 7 days in the ICU	Cost‐Utility Analysis	Trial‐based economic evaluation	ABLE Trial	**Intervention**: Fresher RBCs (stored for ≤ 7 days) **Comparator**: Current standard‐aged RBCs	**Perspective**: NHS and personal social services perspective **Time Horizon**: 1 year **Currency**: UK pounds/ 2015 **Discount**: Not Applicable	Mean incremental cost for fresh blood vs. standard‐aged blood was −£231, with QALYs gained of 0.010. ICER: £23,100	Implementation of preoperative anaemia measures in patients with iron‐deficiency anaemia, undergoing elective surgery, would reduce the risk of death, length of hospital stay, and hospital costs.
Sanal et al. (2023) (Turkey)	Preoperative anaemia management / Surgery (Cardiovascular)	Patients undergoing cardiovascular surgery at Ankara Bilkent City Hospital	Cost‐Effectiveness and Budget Impact Analyses	Model‐based evaluation using real‐world and published data	Single‐centre hospital database (pre/post PBM), literature‐based complication probabilities	**Intervention**: Implementation of a multi‐phase PBM programme **Comparator**: Usual care without PBM	**Perspective**: Turkish Social Security Institution (SSI) **Time horizon**: Hospital stay and 20‐month budget impact **Currency**: TRY / 2023 **Discount**: Not applicable	PBM associated with a 21% reduction in RBC use and 23.7% reduction in all blood products. Savings of 518.68 TRY/patient and 1.6 M TRY from transfusions. Total savings of 8.2 M TRY (€404,725); ICER not calculated but PBM dominated usual care.	PBM implementation reduced transfusions, complications, and total costs. It was budget‐saving and cost‐effective in a cardiovascular surgery setting in Turkey.
Drabinski et al. (2020) (Germany)	Patient Blood Management / Surgery (General elective)	Patients undergoing elective surgery	Cost‐Minimization Analysis	Model‐based evaluation using administrative database	Representative secondary data from the German database DRG Statistic for 2015 (DRG‐Statistic 2015)	**Intervention**: Patient Blood Management Preoperative Anaemia Management (PAMS) **Comparator**: No PAMs	**Perspective**: Healthcare system perspective **Time horizon**: hospital stay duration **Currency**: Euro / 2015 **Discount rate**: Not Applicable	The cost to avoid one transfusion was €831, and to save one RBC unit was €405.	FMC‐based pre‐operative optimisation of haemoglobin is cost‐effective in primary knee arthroplasty and should be considered for iron‐deficiency anaemia patients.
Basora et al. (2018) (Spain)	Intravenous iron / Surgery (Hip/Lower limb arthroplasty)	Iron‐deficient patients undergoing knee arthroplasty	Cost‐Effectiveness Analysis	Observational cohort study	A previous Cohort study [42]	**Intervention**: Intravenous iron—Ferric carboxymaltose administered before surgery **Comparator**: Haemoglobin non‐optimisation	**Perspective**: Hospital **Time horizon**: Length of stay in hospital. **Currency**: Euros / Base year not specified	30‐day ICER: €33,065 saved per additional MACE averted with the restrictive vs. liberal strategy. At 1 year, the cost‐utility ratio favoured the restrictive strategy at €191,500 saved per QALY gained.	In patients with AMI and anaemia, the restrictive transfusion strategy was dominant (cost‐saving and outcome‐improving) at 30 days. At 1 year, the restrictive strategy remained cost‐saving, but clinical non‐inferiority on MACE was no longer maintained.
Durand‐Zaleski et al. (2022) (France & Spain)	Patient Blood Management / Anaemia Cardiology	Acute myocardial infarction (AMI) patients with anaemia	Cost‐Effectiveness Analysis & Cost Utility Analysis	Trial‐based economic evaluation	REALITY Trial conducted in 35 hospitals in France and Spain	**Intervention**: Restrictive transfusion strategy (transfusion triggered by haemoglobin ≤8 g/dL, with a target between 8 and 10 g/dL) **Comparator**: Liberal strategy (transfusion triggered by haemoglobin ≤10 g/dL, with a target >11 g/dL)	**Perspective**: Hospital perspective (France) **Time Horizon**: 30 days, 1year **Currency**: Euro / 2021 **Discount rate**: Both costs and outcomes were undiscounted	30‐day ICER: €33 065 saved per additional MACE averted with the restrictive vs. liberal strategy. At 1 year, the point estimate of the cost‐utility ratio was €191 500 saved per QALY gained in favour of the restrictive strategy.	PBM is cost‐saving for cardiac and non‐cardiac surgery in Turkey.
Irving et al. (2019) (Australia)	Patient Blood Management / Intensive care	Adults with an anticipated ICU stay of at least 24 h when the decision had been made to transfuse at least one RBC unit.	Cost‐Utility Analysis	Trial‐based economic evaluation	TRANSFUSE clinical trial	**Intervention**: Freshest compatible RBC units **Comparator**: Oldest available compatible RBC units	**Perspective**: Healthcare provider perspective **Time horizon**: 6 Months **Currency**: USD dollar/ 2016 **Discount rate**: Not Applicable (due to short‐time horizon)	Unadjusted results suggest short‐term storage is more cost‐effective and slightly improves QALYs than long‐term storage, but these differences are not significant. Even after adjustment, short‐term storage remains dominant with minimal, insignificant effects.	The TRANSFUSE trial shows that short‐term storage of red blood cells (RBCs) does not significantly improve quality of life or reduce costs compared to long‐term storage in critically ill adults, supporting the continued practice of using the oldest available RBCs.
Husk et al. (2024) (USA)	Patient Blood Management / Surgery (Urogynaecology)	Patients undergoing minimally invasive urogynaecology surgery	Cost‐Utility Analysis	Model‐based analysis using administrative data	2020 Medicare Fee Schedule and Hospital Outpatient Prospective Payment System Current Procedural Terminology (CPT) Codes and 2020 Physician Fee Schedules	**Intervention**: SAs (epoetin alfa, beta, theta and zeta and darbepoetin alfa) **Comparator**: Est. supportive care, defined as adjusting cancer treatment, RBCT and iron supplementation	**Perspective**: Hospital perspective **Time horizon**: short‐term **Currency**: USD (2024), Base Year: 2024 **Discount rate**: Not Applicable (due to short‐time horizon)	ICER of routine T&S vs. no T&S: $63,721,632/QALY	Routine preoperative T&S is not cost‐effective for minimally invasive urogynecological surgery
Tatar et al. (2022) (Turkey)	Comprehensive anaemia management (Pillar 1 of PBM) / Cardiac and orthopaedic surgery	Patients undergoing coronary artery bypass grafting (CABG) and hip/knee arthroplasty	Cost‐Effectiveness Analysis and Budget Impact Analysis	Model‐based evaluation using decision‐tree simulation	Meta‐analysis data (Kleinerüschkamp et al.) for adverse event probabilities; Turkish SSI reimbursement costs and expert resource use data	**Intervention**: Preoperative diagnosis and treatment of anaemia using IV ferric carboxymaltose (FCM) **Comparator**: No PBM implementation (standard care)	**Perspective**: Turkish Social Security Institution (SSI) **Time horizon**: 30‐day hospitalisation for cost‐effectiveness; 1‐year for budget impact **Currency**: Turkish Lira / 2021 **Discounting**: 3% for long‐term complications	PBM dominated standard care (fewer adverse events, lower cost): −₺7,504 (non‐cardiac surgery) and −₺6,102 (cardiac surgery) per patient Avoided adverse events: 1768 (non‐cardiac) and 1244 (cardiac)	PBM was a cost‐saving and cost‐effective strategy for major surgery in Turkey. The model showed robust results across sensitivity analyses and highlighted the economic value of anaemia management in surgical pathways.
Linn et al. (2023) (USA)	Pharmacist‐led ESA dosing protocol / Haemodialysis (CKD‐related anaemia)	Hospitalised adult patients with chronic kidney disease receiving haemodialysis and epoetin alfa‐epbx	Cost analysis (retrospective pre–post cohort design)	Multisite retrospective cohort study before–after design)	Hospital EMR and pharmacy data; 6‐month periods before and after protocol implementation	**Intervention**: Pharmacist‐driven consultation for initial ESA (epoetin alfa‐epbx) dosing, including outpatient dose conversion and clinical review **Comparator**: Usual physician‐directed ESA dosing prior to implementation	**Perspective**: Hospital/pharmacy **Time horizon**: 6‐month pre‐ and post‐intervention periods **Currency**: USD ($) / 2021–2022 **Discounting**: Not applied (short‐term)	−$640.42 per patient (38% reduction in acquisition cost, *p* < 0.0001) Dose reduced by 26.2% (*p* = 0.0004); average dose: 13,694 vs. 10,112 units No statistically significant difference in transfusions or adverse events No ICER calculated	Pharmacist‐led ESA dosing protocol reduced ESA acquisition costs and average dosing in hospitalised haemodialysis patients. Results support expanded pharmacist involvement to reduce inappropriate or excessive ESA use and optimise transitions of care.
	Red Blood Cell Transfusion Decision Protocol / General inpatient medicine (non‐bleeding adult patients)	Adult, non‐bleeding inpatients receiving RBC transfusions in a tertiary academic hospital	Cost Analysis (pre‐post design)	Retrospective before–after analysis using hospital and blood bank records	Electronic health records and local transfusion database (6 months pre‐ and post‐intervention)	**Intervention**: Implementation of a 2‐factorial transfusion decision protocol based on haemoglobin level and anaemia symptoms **Comparator**: Usual transfusion practice before protocol introduction	**Perspective**: Hospital/provider **Time horizon**: 6 months before and 6 months after intervention **Currency**: € (converted from Polish złoty using exchange rate of 4.65 PLN/€) **Discounting**: Not applicable (short‐term)	€51,411 total cost savings (56.4% reduction) RBC transfusions reduced from 811 to 394 (−51.4%) Transfusion rate fell from 1.8% to 0.6% Appropriate transfusions increased from 23.6% to 37.4% Inappropriate transfusions still made up 63.6% post‐intervention No ICER calculated	The implementation of a structured transfusion decision protocol significantly reduced inappropriate transfusions, RBC use, and transfusion‐related costs. Despite the improvements, further clinician education is needed to reduce residual inappropriate transfusions. Transfusion‐associated labour and material costs nearly equalled the RBC acquisition cost, underlining the value of optimisation.

Regarding the time horizon, one study [25] adopted a long‐term perspective (lifetime) using a discount rate of 3.5% for costs and outcomes. Two studies [33, 35] did not specify an explicit time horizon. The remaining studies primarily adopted short‐term time horizons, typically focusing on the duration of hospital stay. The focus of the studies’ perspectives included hospitals [26, 29, 32], healthcare services [30, 39], healthcare providers [27], payers [31], social security institutions [31] and broader perspectives such as the NHS and personal social services [25, 28]. One study [33] did not explicitly mention its perspective. The studies utilised various sources of effectiveness data, including randomised clinical trials [27, 28, 32, 36], literature reviews [25, 31, 35, 39], cohort studies [29] and patient databases [26, 30, 33].

### 3.4 Economic Evidence on Anaemia Treatment Strategies

#### 3.4.1 Anaemia in Chronic Diseases and Inherited Disorders

Two studies addressed anaemia in the context of chronic disease [25, 32]. The first, a UK Health Technology Assessment (HTA) [25], evaluated erythropoietin for chemotherapy‐induced anaemia in cancer patients. Based on a systematic review, the study found a QALY gain of 0.07 (CI 95%: −0.278 to 0.433) and a relative risk of transfusion of 0.62 (95% CI: 0.46 to 0.84), implying a 38% reduction in transfusion probability compared to standard care (see Table 5). However, the intervention increased costs differentials per patient by £1829.77 for erythropoietin alpha (rising to £3087.58 for erythropoietin beta), placing its cost‐effectiveness near or above the £20,000–£30,000 per QALY threshold used by NICE. Moreover, there was uncertainty around value for money given the wide confidence intervals reported for QALYs gains (see Table 5).

**TABLE 5:** Table summarising findings of economic evaluations for intervention versus comparator.

Study authors (Year)	Intervention	Comparator (detailed or broken down)	Incremental costs*	Relative risk of transfusion	Incremental blood units	Hospital days	Survival	Incremental quality adjusted Life Years (QALYs)	Hierarchal RRT	Hierarchal blood units	Hierarchal hospital days	Hierarchal survival	Hierarchal QALY
Crathorne et al. (2016)	Darbepoetin alfa	Best supportive care: RBCT [^1^] as needed, iron supplementation, and adjustment of cancer treatment—reflective of UK NHS cancer anaemia protocols.	£2913.02 (95%CI £1642.83 to £4183.21)	RR 0.62 (95%CI 0.46 to 0.84)	−0.87 (95% CI: −1.28 to −0.46)	NA	HR (Survival): 0.967 (95%CI: 0.81 to 1.12)	0.07 (95% CI −0.278 to 0.433)	Consider: ↑Cost, ↑Effect	Consider: ↑Cost, ↑Effect		Weak evidence**: ↑Cost, ↑Effect	Weak evidence**: ↑Cost, ↑Effect
Crathorne et al. (2016)	Erythropoietin alpha	As above—standard care involving RBCT and supportive treatment	£1829.77 (95% CI £980.50 to 2679.04)	Same as above	Same as above	NA	Same as above	Same as above	Consider: ↑Cost, ↑Effect	Consider: ↑Cost, ↑Effect		Weak evidence**: ↑Cost, ↑Effect	Weak evidence**: ↑Cost, ↑Effect
Crathorne et al. (2016)	Erythropoietin beta	As above—standard care involving RBCT and supportive treatment	£3087.58 (95%CI £1734.44 to £4439.48	Same as above	Same as above	NA	Same as above	Same as above	Consider: ↑Cost, ↑Effect	Consider: ↑Cost, ↑Effect		Weak evidence**: ↑Cost, ↑Effect	Weak evidence**: ↑Cost, ↑Effect
Crathorne et al. (2016)	Erythropoietin theta	As above—standard care involving RBCT and supportive treatment	£1849.57 (95% CI £1022.59 to £2677.80)	Same as above	Same as above	NA	Same as above	Same as above	Consider: ↑Cost, ↑Effect	Consider: ↑Cost, ↑Effect		Weak evidence**: ↑Cost, ↑Effect	Weak evidence**: ↑Cost, ↑Effect
Crathorne et al. (2016)	Erythropoietin zeta	As above—standard care involving RBCT and supportive treatment	£1,869.38 (95% CI £891.36 to £2,784.27)	Same as above	Same as above	NA	Same as above	Same as above	Consider: ↑Cost, ↑Effect	Consider: ↑Cost, ↑Effect		Weak evidence**: ↑Cost, ↑Effect	Weak evidence**: ↑Cost, ↑Effect
Durand‐Zaleski et al. (2022)	Restrictive RBC transfusion (Hb ≤8 g/dL, post‐Hb 8–10 g/dL)	Liberal transfusion strategy (Hb ≤10 g/dL, post‐Hb ≥11 g/dL); transfusions administered at higher Hb threshold—AMI patients in cardiology units	−£1149 (95% CI: −£1426.67 to £3830.24)	NA	−0.4 units (95% CI: −0.62 to −0.18)	Ward: ‐0.7 days (95% CI: −2.14 to 0.74)	(% diff survival) −3.0% (95% CI: −8.4% to 2.4%)	0.006 (95% CI −0.056 to 0.046)		Accept: ↓Cost, ↑Effect	Weak evidence**: ↓Cost, ↑Effect	Weak evidence**↓Cost, ↑Effect	Weak evidence**↓Cost, ↑Effect
Irving et al. (2019)	Short‐term stored RBCs (freshest compatible unit)	Standard issue RBCs (oldest compatible units within expiry window); usual blood bank practice to minimise wastage – ICU adult population	−£1838.12 (95% CI: −£4354.42 to £554.24)	NA	0.1 units (95% CI: −0.3 to 0.4)	Ward: ‐1.0 days (95%CI −2.1 to 0.9)	(% diff survival) 0.4% (95% CI: −2.1% to 3.0%)	0.003 (95% CI: −0.003 to 0.008)		Accept: ↓Cost, ↑Effect	Weak evidence**↓Cost, ↑Effect	Weak evidence**↓Cost, ↑Effect	Weak evidence**↓Cost, ↑Effect
Walsh et al. (2017)	Fresh RBCs (stored ≤7 days)	Standard NHS blood service issue (oldest compatible RBCs, ∼21 days average) – critically ill patients in ICU	−£278.61 (95%CI 5881.0 to 5325.0)	NA	0.40 (95%CI NA)	0.70 (95%CI NA)	NA	−0.01 (95% CI −0.078 to 0.057)		Accept: ↓Cost, ↑Effect	Consider: ↓Cost, ↓Effect		Weak evidence**: ↓Cost, ↓Effect
Bedair et al. (2015)	Erythropoietin alpha pre‐op for THA/TKA patients with Hb <13 g/dL	No EPO; transfusion given if postoperative Hb <10 g/dL and symptomatic – orthopaedic care following standard hospital policy	£288.40 (CI95% NA)	(Diff in %) −41 (95% CI: −58% to −24%)	−1.6 units (95%CI NA)	−0.30 days (95% CI: −1.65 to 1.05 days)	NA	NA	Consider: ↑Cost, ↑Effect	Weak evidence**: ↑Cost, ↑Effect	Weak evidence**: ↑Cost, ↑Effect		
Şanal et al. (2024)	Multiphase Patient Blood Management (PBM) programme for cardiovascular surgery	Standard perioperative care prior to PBM implementation at a large tertiary hospital	−£82.83 (transfusion costs only) −£417.34 (total saving)	RR 0.71 (based on reduction from 55.3% to 39.1%)	−0.88 RBC units per patient	NA	NA	NA	Weak evidence**: ↓Cost, ↓Effect	Weak evidence**: ↓Cost, ↓Effect			
Basora et al. (2018)	Intravenous iron	No pre‐op iron supplementation: transfusions as needed	£898.41 (CI95% NA)	RR of transfusion: 0.335 (95%CI NA)	−0.9 units (95%CI NA)	NA	NA	NA		Weak evidence**: ↑Cost, ↑Effect	Weak evidence**: ↑Cost, ↑Effect		
Meybohm et al. (2020)	Multimodal PBM (iron therapy, tranexamic acid, cell salvage)	Conventional care without coordinated PBM; no standardised use of iron therapy or intra‐op blood‐saving strategies	−£160.0 (CI95% NA)	RR of transfusion: 0.61 (95% CI: 0.55 to 0.68)	−0.43 units (95% CI: −0.54 to −0.31)	−0.45 days (95% CI: −0.65 to −0.25 days)	NA	NA	Accept: ↓Cost, ↑Effect	Accept: ↓Cost, ↑Effect	Accept: ↓Cost, ↑Effect		
Trentino et al. (2021)	Routine preoperative Type & Screen (T&S) before elective surgery	No routine T& S; emergency transfusion with uncrossmatched O‐negative blood when needed	−£1,540.30 (95%CI −£2,821.66 to −£259.06)	(Diff in %) −6.5% (95% CI: −11.3 to −1.7)	NA	−1.4 days (95% CI: −3.02 to 0.22)	NA	NA	Accept: ↓Cost, ↑Effect		Weak evidence**: ↓Cost, ↑Effect		
Drabinski et al. (2020)	Preoperative PBM targeting IDA (iron deficiency anaemia) patients	Conventional pre‐op care with no systematic anaemia screening or iron supplementation—reflective of typical German hospital care prior to PBM uptake	−£1,862.48 (CI95% NA)	NA	NA	−2.37 (95%CI NA)	(% diff survival) −0.01 (95%CI NA	NA			Weak evidence**: ↓Cost, ↑Effect	Weak evidence**: ↓Cost, ↑Effect	
Drabinski et al. (2020)	Preoperative PBM targeting for non‐IDA patients	Conventional pre‐op care with no systematic anaemia screening or iron supplementation—reflective of typical German hospital care prior to PBM uptake	−£106.49 (CI95% NA)	NA	NA	−0.12 (95%CI NA)	(% diff survival) 0.00 (95%CI NA	NA			Weak evidence**: ↓Cost, ↑Effect	Weak evidence**: ↓Cost, ↑Effect	
Husk et al. (2024)	Preoperative T&S before minimally invasive urogynecological surgery	No preoperative T& S; use of O‐negative emergency blood in rare cases requiring transfusion	£10.35 (CI95% NA)	NA	NA	NA	NA	<0.00001 (CI95% NA)					Reject: ↑Cost, = Effect

*Note*: *—Adjusted for inflation (year = 2022) and for Purchasing Power Parity; Cost: ↑ Higher: Increased cost relative to the comparator; Cost: ↓ Lower: Reduced cost relative to the comparator; Effectiveness: ↑ Higher: More effective compared to the comparator; Effectiveness: ↓ Lower: Less effective compared to the comparator.

^a^ [1]RBCT—Red blood cell transfusion: **Weak evidence—Indicates that results were not statistically significant (e.g., confidence intervals crossing the null) or that no statistical testing was reported in the publication.

The second study [32], a cost‐utility analysis conducted in France and Spain, compared restrictive versus liberal transfusion strategies in anaemic patients with acute myocardial infarction (AMI), based on SIT trial [37] data (*n* = 648). The restrictive approach was associated with uncertain cost savings per patient of −£1,149 (95% CI: −£1426.67 to £3830.24) and reduction in hospital stay of −0.7 days (95% CI: −2.14 to 0.74). QALY gains were also minimal and uncertain, 0.006 (95% CI: −0.056 to 0.046), while transfused units decreased by −0.4 units (95% CI: −0.62 to −0.18) (see Table 5). These findings align with those of the MINT trial, which showed no significant difference in 30‐day mortality between strategies [38, 39]. The study highlights that the restrictive strategy may offer modest cost savings and efficiency in short‐term patient management, but the residual risks and uncertain long‐term effectiveness highlight the need for further research.

#### 3.4.2 Management of Anaemia in ICU Settings

Anaemia affects about 66% of ICU patients, making it the most common hematologic condition in this setting [40]. Two of the selected studies [27, 28] focused on anaemia treatments during ICU hospitalisation.

The first, a UK‐based HTA [28], compared fresher red blood cells (RBCs) (stored ≤7 days) with standard‐aged RBCs in critically ill patients (*n* = 100 per group). There were uncertainties around the differences for the fresher RBC strategy, both in terms of costs, −£278.61 (95%CI 5881.0 to 5325.0), and improvement in QALYs, −0.01 (95%CI −0.078 to 0.057) (see Table 5). As such, the intervention was deemed less likely to be cost‐effective.

The second, an Australian cost‐utility analysis [27], based on the TRANSFUSE trial [41], evaluated short‐term versus long‐term RBC storage in 4994 ICU patients. Transfusing short‐term stored RBCs yielded uncertainty in both cost savings per patient (AUD −2,358; −£1,838.12 [95% CI: −£4,354.42 to £554.24]) and QALYs, 0.003 (95% CI: −0.003 to 0.008) (see Table 5). These uncertainties limited the strength of evidence for its cost‐effectiveness.

#### 3.4.3 Preoperative and Postoperative Anaemia Management

Perioperative anaemia is associated with increased complications and longer hospital stays. Seven studies evaluated preoperative anaemia management interventions [30, 31, 33, 35, 29, 39].

A Spanish observational CEA study [29] using cohort data [42] on 52 patients undergoing knee arthroplasty reported that preoperative optimisation with intravenous ferric carboxymaltose (FCM) reduced transfusion rates by −0.41 units (95% CI: −0.58 to −0.24) but increased costs by $348 (£288.40 [95% CI NA]) (see Table 5). While clinical outcomes, such as relative risk of transfusion (RR = −0.41 [95% CI: −0.58 to −0.24]) improved, the absence of QALY data limited the assessment of cost‐effectiveness (see Table 5).

Similarly, a US CMA study [33] found that preoperative erythropoietin alpha reduced transfusion needs by 41.0 percentage points (95% CI: −58.0% to −24.0%) in patients undergoing THA or TKA, though it increased costs by $303.80 (adjusted £288.40; [CI95% NA]) per patient. Again, QALYs were not reported.

A Turkish CEA [31] reported savings for orthopaedic and CABG surgeries following intravenous iron treatment, with fewer adverse events and reduced need for transfusion (see Table 5). However, the study also lacked statistical comparisons and QALY data [31].

An Australian CEA study [30] involving 680 patients undergoing elective colorectal surgery found preoperative anaemia screening and FCM use, reduced risk of transfusion by 6.5% (95% CI: −11.3 to −1.7), with a cost saving of AUS $2,974.0 (−£1,540.30 [95%CI −£2,821.66 to −£259.06]). Again, the overall cost‐effectiveness could not be determined due to missing QALY data.

By using representative secondary data from the German DRG database, a CMA study [39] highlighted €1849.26 (−£1,862.48; CI95% NA) in savings through reduced transfusions, lower mortality and shorter stays. Similarly, a German CBA study [35] utilised findings from a meta‐analysis, which reported that PBM's widespread implementation— including the diagnosis of preoperative anaemia, treatment with intravenous iron, cell salvage and rational transfusion—reduced transfusions (RR 0.61 [95% CI: 0.55 to 0.68]), decreased blood use by 0.43 units (95% CI: −0.54 to −0.31) per patient, shortened hospital stays by an average of 0.45 days (CI95% NA) per patient, and achieved €105.73 (£106.49; [CI95% NA]) in savings (see Table 5). Again, despite these promising clinical and economic benefits, further data and analyses are required to evaluate long‐term cost‐effectiveness.

Finally, a US cost‐effectiveness analysis in urogynecological surgery assessed routine preoperative type and screen (T&S) versus no T&S using O‐negative blood. The base case assumed a 1.26% transfusion probability and a transfusion reaction rate of 0.0013% with T&S versus 0.4% without it. With a negligible QALY gain (<0.00001 [CI95% NA]) and an added cost of $13.40 (£10.35; CI95% NA), T&S was not cost‐effective (see Table 5). Sensitivity analysis showed cost‐effectiveness only if the transfusion reaction risk exceeded 12%, far above reported rates.

### 3.5 Economic and Clinical Evidence From the Hierarchical Decision Matrix

The Hierarchical Decision Matrix (HDM) (see Table 5 and Figure S1) provided a structured framework to assess the cost‐effectiveness of anaemia management strategies, incorporating clinical and economic outcomes such as relative risk of transfusion (RRT), incremental blood use, hospital length of stay, costs, mortality rates and QALYs. Given the marked heterogeneity in interventions, study populations and outcome reporting, the HDM approach facilitated a more consistent comparison of findings across a diverse set of studies, while also highlighting important gaps and inconsistencies across studies. [43, 44, 45]

Restrictive transfusion strategies consistently emerged as cost‐saving interventions, reducing blood use and hospital stay without compromising patient outcomes. Two of the selected studies [32, 35] demonstrated cost savings and lower transfusion risks, reinforcing the endorsement of restrictive strategies. However, uncertainty remains in acute myocardial infarction (AMI) patients, where conflicting evidence from the REALITY [46] and MINT trials [38, 39] suggests that some subgroups may benefit from a more liberal approach.

ESAs consistently yielded higher costs with limited clinical benefits. Erythropoietin therapies such as alpha, beta, theta and zeta variants were associated with substantial per‐patient cost increases (ranging from £1829 to £3087) [25] and only modest reductions in transfusion risk (e.g., RR 0.62, 95%CI: 0.46–0.84). Gains in QALYs were small (0.00 to 0.07) and subject to wide uncertainty. Given their high acquisition cost and limited impact on long‐term outcomes, ESAs are unlikely to be cost‐effective as a routine strategy for anaemia management in most settings.

Iron supplementation showed mixed evidence on cost‐effectiveness. While oral iron has previously been associated with cost savings (as reported in NICE NG24) [47], results for intravenous iron were variable. One study reported increased costs (£1064 per patient) [29] despite reduced transfusion rates, while others demonstrated modest savings and fewer transfusions in perioperative and surgical settings. Evidence from recent evaluations [30, 31] suggests that iron therapy may form a valuable component of multimodal anaemia management, yet the lack of robust QALY data and inconsistent economic outcomes limits firm conclusions on long‐term cost‐effectiveness.

Fresh versus standard RBC transfusion strategies showed no clear cost or clinical advantage. While one study [27] reported minor cost savings with fresher RBCs, another [28] found higher costs and longer hospital stays. Given this, standard RBC storage policies remain the more pragmatic and cost‐neutral strategy for most healthcare systems.

Patient Blood Management (PBM) strategies, particularly preoperative anaemia screening and treatment, emerged as the most promising approach in most studies. Selected papers [31, 35, 39] demonstrated cost savings and reduced transfusion rates, making PBM a potentially cost‐effective alternative to routine transfusion. However, while transfusion risk and resource use were consistently reduced, most PBM studies lacked QALY estimates, which prevented a full economic evaluation against international willingness‐to‐pay thresholds. Future research should therefore prioritise standardised utility measurement and modelling of long‐term outcomes.

Several entries in the HDM matrix are marked as ‘weak evidence’ (see Table 5), suggesting non‐significant results, a lack of formal testing, or reliance on uncontrolled before‐and‐after and cohort studies. While such studies may suggest promising trends (e.g., reduced transfusion rates or costs) or lower costs, they are limited by confounding, selection bias or short‐term horizons. They should be considered with caution and serve primarily to highlight areas that require more rigorous evaluation, rather than providing definitive evidence of cost‐effectiveness.

## 4 Discussion

This review systematically assesses recent economic and clinical evidence on anaemia treatments, including blood transfusions, addressing gaps highlighted by previous narrative reviews. Our analysis specifically focused on studies from OECD countries published since the last NICE blood transfusion guideline NG24 in 2015, enhancing its direct relevance to UK healthcare. In addition, to further improve clarity and applicability, included studies were assessed using the NICE checklist for UK relevance. The review thus identifies newly published studies, summarises findings in a structured and policy‐relevant manner, and clearly highlights evidence gaps to guide future healthcare decisions both from the UK setting and broader.

A total of 11 studies were identified and analysed as relevant for the anaemic population. While the literature has expanded, the findings overall present a mixed picture regarding cost‐effectiveness. Some interventions showed clinical benefits and potential cost savings, whereas others resulted in higher costs without clear evidence of improved outcomes. Furthermore, the absence of QOL data and comprehensive CUAs limits the ability to draw definitive conclusions. This underscores the need for further research to evaluate the long‐term economic and societal impacts of these.

For example, erythropoietin for chemotherapy‐induced anaemia modestly improved QOL and reduced transfusion needs, but it increases costs substantially (£1829.77 per patient), and a lack of evidence on survival benefits limits its cost‐effectiveness assessment. While ESAs [25] may benefit select populations, their high costs and risks (e.g., thromboembolic events) necessitate cautious implementation aligned with cost‐effectiveness thresholds.

A number of identified studies included economic evaluations of different transfusion thresholds [13]. Most studies found no significant benefit in using higher haemoglobin thresholds for transfusion, supporting the case for lower thresholds. Some trials also noted economic advantages, such as reduced hospital costs and transfusion dependency. For example, the REALITY trial on acute myocardial infarction patients demonstrated that thresholds are clinically non‐inferior to liberal strategies while achieving an 84% probability of cost savings [32]. However, a later trial (MINT) reported a possible benefit for higher thresholds in this population [48].

In addition, using blood transfusions to prevent silent cerebral infarcts in sickle cell anaemia (SCA) patients, while increasing costs, resulted in reduced transfusion requirements and shorter hospital stays. These long‐term benefits may suggest potential cost‐effectiveness despite the higher initial costs, but it depends on confirming the improvement in the outcome. These findings indicate a need for further research into alternative therapies and long‐term outcomes for chronic anaemia management.

Managing anaemia in ICU patients poses unique challenges, especially regarding the use of fresher RBCs. This review indicates that while some studies show minor outcome improvements with fresher RBCs, the benefits do not justify the increased costs. The selected studies [27, 28] indicate that fresher RBCs do not significantly improve outcomes or cost‐effectiveness. These findings suggest that fresher or short‐term storage RBCs may not be a cost‐effective strategy for critically ill patients, emphasising the need for further research into alternative treatments.

Perioperative anaemia is a known risk factor for higher transfusion needs, complications and longer hospital stays [49]. Improving haemoglobin levels may improve outcomes. Our selected studies found that preoperative intravenous iron therapy reduced transfusion rates and improved QALYs in knee arthroplasty patients despite higher initial costs [29]. In contrast, we also noted that while preoperative erythropoietin alpha lowered postoperative transfusion needs, it was not cost‐saving and lacked QALY data for cost‐effectiveness analysis [33]. These studies highlight the potential benefits of preoperative interventions, although the economic advantages remain unclear.

PBM strategies, including preoperative screening and treatment of anaemia [30, 31, 35, 39] (using intravenous FCM [30, 31, 35, 39], rational transfusion [35], and cell salvage) [35], and routine preoperative type and routine transfusion in urogynecological surgery and routine transfusion if needed [26], have shown mixed evidence regarding their cost‐effectiveness in managing perioperative anaemia, though some studies promise cost savings alongside clinical benefits. Selected studies [30, 31, 34, 35] demonstrated the economic and clinical benefits of PBM. While PBM strategies promise to improve clinical outcomes, all studies highlighted cost savings and demonstrated potential for cost‐effectiveness.

Our findings align with those of a previous literature review [50]. The network meta‐analysis of 393 RCTs found that PBM interventions reduced transfusion rates but showed no effect on key clinical outcomes such as mortality or major morbidity and offered limited evidence of cost‐effectiveness.

### 4.1 Alignment With NICE Guidelines: Key Insights

The findings from this review generally align with several key recommendations from the NICE NG24 guideline [13]. However, gaps remain in the economic evidence, particularly for certain patient groups and emerging interventions.

*Restrictive Transfusion Strategies*: Our evidence reaffirmed NICE NG24's recommendation of the cost‐effectiveness of restrictive transfusion thresholds, showing that using lower haemoglobin triggers can reduce costs without compromising clinical outcomes [13]. However, NICE may yet consider guidance on the optimal transfusion threshold for AMI patients, as conflicting findings from the REALITY and MINT trials suggest that some patient subgroups may benefit from a more liberal approach [32].

*Erythropoiesis‐Stimulating Agents (ESAs)*: NICE does not currently recommend ESAs for routine anaemia management due to concerns about high costs and limited cost‐effectiveness [13]. The findings from this review reinforce this standpoint, indicating that while erythropoietin may reduce transfusion needs, its high costs and uncertainty around QALY benefits make it less likely to be a cost‐effective solution in these cases [25].

*Iron Supplementation*: NICE endorses preoperative iron therapy, particularly FCM, for managing anaemia before surgery [13]. This review supports that recommendation but also highlights that definitive cost‐effectiveness data remain limited. [29] Further economic evaluations are needed to determine whether intravenous iron consistently improves long‐term outcomes and reduces transfusion dependency.

*Fresh vs. Standard RBCs and other PBM interventions*: Current NICE guidance does not distinguish between fresher and standard‐aged RBCs for transfusions [13]. The findings of this review suggest that fresher RBCs offer no significant cost‐effectiveness advantage, reinforcing the current NICE position that standard‐aged RBCs are equally effective and more economical. While PBM interventions (i.e., incorporating strict transfusion thresholds and careful blood component management throughout all surgical phases) demonstrated cost savings in cardiovascular surgeries, their overall cost‐effectiveness remains uncertain. In contrast, an evaluation of routine preoperative type and screen (T&S) in urogynecological surgery found that lowering transfusion and transfusion reaction rates does not make universal preoperative T&S cost‐effective. Hence, further research is needed to assess its long‐term economic and clinical impact.

### 4.2 Limitations

This review offers important insights into the economic evaluations of anaemia management interventions, but it also has several limitations. The number of UK‐specific studies is limited, which may not accurately reflect the nuances of the NHS system. However, the inclusion of international studies provides valuable broader perspectives.

Many studies have small sample sizes, which affects the generalizability of the findings. The absence of confidence intervals in some studies made it difficult to conduct a robust meta‐analysis, leading us to rely on descriptive assessments instead.

Another key limitation of this review is the inconsistent reporting of QALYs and ICERs across included studies. While some evaluations provided robust cost‐utility analyses, many reported only intermediate outcomes (e.g. transfusion rates, hospital costs) without linking them to utility gains. This limited the ability to apply standard cost‐effectiveness thresholds and restricted cross‐study comparability. In addition, the exclusion of low‐quality studies from synthesis—though methodologically justified—may have reduced the breadth of evidence considered.

Furthermore, most studies focus on short‐term outcomes, such as hospital stay duration or immediate transfusion needs, which overlook potential long‐term benefits. To enhance the understanding of cost‐effectiveness, further research is needed, particularly in the UK, which includes long‐term economic evaluations and considers QoL outcomes and a wider range of perspectives.

Despite these limitations, this review provides valuable insights that can inform decision‐making in anaemia management. Addressing these concerns in future studies will help strengthen the evidence base in this area.

## 5 Conclusion

The results of this review highlight the potential of cost‐effective alternatives to blood transfusions, such as restrictive transfusion strategies and most patient blood management strategies, including anaemia treatment and strict transfusion thresholds, to optimise anaemia care. These interventions can reduce transfusion dependency, improve patient outcomes and generate cost savings, aligning with and extending UK national guidelines.

However, gaps remain in economic evaluations, particularly in long‐term outcomes, productivity costs and UK‐specific data. Future research should incorporate a wider healthcare perspective and would benefit from the use of a standardised framework for assessing anaemic management to allow reliable comparisons to be drawn. Addressing these gaps is essential to guide policy and ensure sustainable, effective anaemia management strategies within the NHS. Integrating evidence‐based approaches will enhance resource utilisation and patient care across diverse populations.

## Author Contributions

H. Farabi and F. Tomini contributed to the study selection, data extraction, analysis, and drafting of the paper. All authors contributed to the further development and refinement of the final version.

## Ethics Statement

The authors have nothing to report.

## Consent

The authors have nothing to report.

## Conflicts of Interest

The authors declare no conflicts of interest.

## Supporting information




**Supporting Fig A1**: Permutation plots summarising findings of economic evaluations for intervention vs. comparator (numbers in cells are the number of studies relevant to each permutation).

## Data Availability

The data supporting the findings of this study are available from the corresponding author upon reasonable request.
