# Supplementary material for: Evaluating Economic and Clinical Impacts of Anaemia Management Strategies: A Systematic Review of the Evidence From the UK Perspective
Source: EJHaem. 2025 Aug 26;6(4):e70124. doi: 10.1002/jha2.70124 (PMC12378652; doi:10.1002/jha2.70124)
Supplement: Supplementary file 1 — Supporting Fig A1: Permutation plots summarising findings of economic evaluations for intervention vs. comparator (numbers in cells are the number of studies relevant to each permutation). [file JHA2-6-e70124-s001.docx]

**Figure A1**. Permutation plots summarising findings of economic evaluations for intervention vs. comparator (numbers in cells are the number of studies relevant to each permutation)

|  |  | **Erythropoietin alpha** | | |  | **Iron therapy** | | |  | **RBC protocol** | | |  | | **Patient Blood Management** | | |
| --- | --- | --- | --- | --- | --- | --- | --- | --- | --- | --- | --- | --- | --- | --- | --- | --- | --- |
|  |  | *Costs* | | |  | *Costs* | | |  | *Costs* | | |  | | *Costs* | | |
|  |  | ↓ | 0 | ↑ |  | ↓ | 0 | ↑ |  | ↓ | 0 | ↑ |  | ↓ | | 0 | ↑ |
| **QALYs** | ↓ | - | - | - |  | - | - | - |  | - | - | - |  | - | | - | 1^f [1]^ |
|  | 0 | - | - | - |  | - | - | - |  | - | - | - |  | - | | - | - |
|  | ↑ | - | - | 2 ^a [2, 3]^ |  | - | - | 1^b [4]^ |  | 1 ^c [5]^ | - |  |  | 4 ^de [6-8]^ | | - | - |
|  |  |  |  |  |  |  |  |  |  |  |  |  |  |  | |  |  |
| **RR of Transfusion** | ↓ | - | - | - |  | - | - | - |  | - | - | - |  | - | | - | - |
|  | 0 | - | - | - |  | - | - | - |  | - | - | - |  | - | | - | - |
|  | ↑ |  | - | 1^a [3]^ |  | - | - | 1^b [4]^ |  | 3^cd [5, 7, 8]^ | - | - |  | 4^de [7-9]^ | | - | - |
|  |  |  |  |  |  |  |  |  |  |  |  |  |  |  | |  |  |
| **Incr. Blood Units** | ↓ | - | - | - |  | - | - | - |  | - | - | - |  | - | | - | - |
|  | 0 | - | - | - |  | - | - | - |  | - | - | - |  | - | | - | - |
|  | ↑ | - | - | 1^a [3]^ |  | - | - | 1^b [4]^ |  | 3^cd [5, 7, 8]^ | - | - |  | 3^de [6-8]^ | | - | - |
|  |  |  |  |  |  |  |  |  |  |  |  |  |  |  | |  |  |
| **Hospital Days** | ↓ | - | - | - |  | - | - | - |  | - | - | - |  | - | | - | - |
|  | 0 | - | - | - |  | - | - | - |  | - | - | - |  | - | | - | - |
|  | ↑ | - | - | 1^a [3]^ |  | - | - | - |  | 2^cd [7, 8]^ | - | 1^d [10]^ |  | 4^f [6-8]^ | | - | - |
|  |  |  |  |  |  |  |  |  |  |  |  |  |  |  | |  |  |
| **Mortality** | ↓ | - | - | - |  | - | - | - |  | - | - | - |  | - | | - | - |
|  | 0 | - | - | - |  | - | - | - |  | - | - | - |  | - | | - | - |
|  | ↑ | - | - | 1^a [3]^ |  | - | - | - |  | 2^cd^ ^[5, 11]^ | - | - |  | 3^def [1, 6, 9]^ | | - | - |

| - **Effectiveness**: - ↑ **Higher**: Better effectiveness compared to the comparator. - 0 Same: Same effectiveness as the comparator. - ↓ **Lower**: Lower effectiveness compared to the comparator. | - **Cost:** - ↑ **Higher:** Increased cost relative to the comparator. - 0 **Same:** No difference in cost relative to the comparator. - ↓ **Lower:** Reduced cost relative to the comparator. |
| --- | --- |
| - **Decision:**  \|  \| - **Treatment strongly rejected** \| \| --- \| --- \| \|  \| - **Treatment less favoured** \| \|  \| - **No obvious decision (look at ICERs, consider other factors)** \| \|  \| - **Treatment more favoured** \| \|  \| - **Treatment strongly accepted** \| \|  \| - **Weak evidence – Indicates that results were not statistically significant (e.g., confidence intervals crossing the null) or that no statistical testing was reported in the publication** \| | |

Note: ^a^ - Comparator: No ESA; Current supportive care with RBCT, iron, and treatment adjustments per clinical judgment or local guidelines.; ^b^ – Comparator: Current practice: no IV iron, no haemoglobin optimisation before surgery; ^c^ – Comparator: Liberal transfusion: (e.g., transfuse at Hb ≤10 g/dL, target Hb ≥11 g/dL); ^d^ – Comparator: RBCT with oldest available compatible units (mean storage ~22 days); ^e^ – Comparator: No PBM, no routine detection or treatment of iron-deficiency anaemia or pre-op anaemia screening: ^f^ – Comparator: No routine T&S, uncrossmatched type O-neg.

**References**

1. Husk, K.E., et al., *Is Preoperative Type and Screen High-value Care? A Cost-effectiveness Analysis of Performing Preoperative Type and Screen Prior to Urogynecological Surgery.* Int Urogynecol J, 2024. **35**(4): p. 781-791.

2. Bedair, H., et al., *Preoperative erythropoietin alpha reduces postoperative transfusions in THA and TKA but may not be cost-effective.* Clinical Orthopaedics and Related Research®, 2015. **473**(2): p. 590-596.

3. Crathorne, L., et al., *The effectiveness and cost-effectiveness of erythropoiesis-stimulating agents (epoetin and darbepoetin) for treating cancer-treatment induced anaemia (including review of TA142): a systematic review and economic model.* 2016.

4. Basora, M., et al., *Should all patients be optimized to the same preoperative hemoglobin level to avoid transfusion in primary knee arthroplasty?* Vox sanguinis, 2014. **107**(2): p. 148-152.

5. Durand-Zaleski, I., et al., *Economic evaluation of restrictive vs. liberal transfusion strategy following acute myocardial infarction (REALITY): trial-based cost–effectiveness and cost–utility analyses.* European Heart Journal-Quality of Care and Clinical Outcomes, 2023. **9**(2): p. 194-202.

6. Drabinski, T., et al., *Estimating the Epidemiological and Economic Impact of Implementing Preoperative Anaemia Measures in the German Healthcare System: The Health Economic Footprint of Patient Blood Management.* Adv Ther, 2020. **37**(8): p. 3515-3536.

7. Meybohm, P., et al., *Health economics of Patient Blood Management: a cost‐benefit analysis based on a meta‐analysis.* Vox Sanguinis, 2020. **115**(2): p. 182-188.

8. Trentino, K., et al., *Screening and treating pre‐operative anaemia and suboptimal iron stores in elective colorectal surgery: a cost effectiveness analysis.* Anaesthesia, 2021. **76**(3): p. 357-365.

9. Şanal, L., S. Günaydın, and M. Tatar, *Cost-Effectiveness and Budget Impact Analyses of Patient Blood Management in a Cardiovascular Surgery Department at Ankara Bilkent City Hospital in Turkey.* Advances in Therapy, 2024. **41**(2): p. 716-729.

10. Walsh, T.S., et al., *The Age of BLood Evaluation (ABLE) randomised controlled trial: description of the UK-funded arm of the international trial, the UK cost-utility analysis and secondary analyses exploring factors associated with health-related quality of life and health-care costs during the 12-month follow-up.* Health Technol Assess, 2017. **21**(62): p. 1-118.

11. Irving, A., et al., *Fresh Red Cells for Transfusion in Critically Ill Adults: An Economic Evaluation of the Standard Issue Transfusion Versus Fresher Red-Cell Use in Intensive Care (TRANSFUSE) Clinical Trial.* Crit Care Med, 2019. **47**(7): p. e572-e579.
